# Investigation and modulation of interleukin-6 following subarachnoid hemorrhage: targeting inflammatory activation for cerebral vasospasm

**DOI:** 10.1186/s12974-022-02592-x

**Published:** 2022-09-16

**Authors:** Brandon Lucke-Wold, William Dodd, Kartik Motwani, Koji Hosaka, Dimitri Laurent, Melanie Martinez, Victoria Dugan, Nohra Chalouhi, Noelle Lucke-Wold, Arnav Barpujari, Christina von Roemeling, Chenglong Li, Richard D. Johnson, Brian Hoh

**Affiliations:** 1 Department of Neurosurgery, University of Florida, Gainesville, FL USA; 2 Department of Physiologic Sciences, University of Florida, Gainesville, FL USA; 3 Department of Radiology, Neuroradiology Division, University of Florida, Gainesville, FL USA; 4 Department of Medicinal Chemistry, University of Florida, Gainesville, FL USA

**Keywords:** Interleukin-6, Subarachnoid hemorrhage, Vasospasm, Microglia activation, p-STAT3

## Abstract

**Background:**

Cerebral vasospasm (CV) can contribute to significant morbidity in subarachnoid hemorrhage (SAH) patients. A key unknown is how CV induction is triggered following SAH.

**Methods:**

Human aneurysmal blood and cerebral spinal fluid were collected for evaluation. To confirm mechanism, c57/bl6 wild type and c57/bl6 IL-6 female knockout (KO) mice were utilized with groups: saline injected, SAH, SAH + IL-6 blockade, SAH IL-6 KO, SAH IL-6 KO + IL-6 administration, SAH + p-STAT3 inhibition. Dual-labeled microglia/myeloid mice were used to show myeloid diapedesis. For SAH, 50 μm blood was collected from tail puncture and administered into basal cisterns. IL-6 blockade was given at various time points. Various markers of neuroinflammation were measured with western blot and immunohistochemistry. Cerebral blood flow was also measured. Vasospasm was measured via cardiac injection of India ink/gelatin. Turning test and Garcia’s modified SAH score were utilized. *P* < 0.05 was considered significant.

**Results:**

IL-6 expression peaked 3 days following SAH (*p* < 0.05). Human IL-6 was increased in aneurysmal blood (*p* < 0.05) and in cerebral spinal fluid (*p* < 0.01). Receptor upregulation was periventricular and perivascular. Microglia activation following SAH resulted in increased caveolin 3 and myeloid diapedesis. A significant increase in BBB markers endothelin 1 and occludin was noted following SAH, but reduced with IL-6 blockade (*p* < 0.01). CV occurred 5 days post-SAH, but was absent in IL-6 KO mice and mitigated with IL-6 blockade (*p* < 0.05). IL-6 blockade, and IL-6 KO mitigated effects of SAH on cerebral blood flow (*p* < 0.05). SAH mice had impaired performance on turn test and poor modified Garcia scores compared to saline and IL-6 blockade. A distinct microglia phenotype was noted day 5 in the SAH group (overlap coefficients *r* = 0.96 and *r* = 0.94) for Arg1 and iNOS, which was altered by IL-6 blockade. Day 7, a significant increase in toll-like receptor 4 and Stat3 was noted. This was mitigated by IL-6 blockade and IL-6 KO, which also reduced Caspase 3 (*p* < 0.05). To confirm the mechanism, we developed a p-STAT3 inhibitor that targets the IL-6 pathway and this reduced NFΚB, TLR4, and nitrotyrosine (*p* < 0.001). Ventricular dilation and increased Tunel positivity was noted day 9, but resolved by IL-6 blockade (*p* < 0.05).

**Conclusion:**

Correlation between IL-6 and CV has been well documented. We show that a mechanistic connection exists via the p-STAT3 pathway, and IL-6 blockade provides benefit in reducing CV and its consequences mediated by myeloid cell origin diapedesis.

**Graphical abstract:**

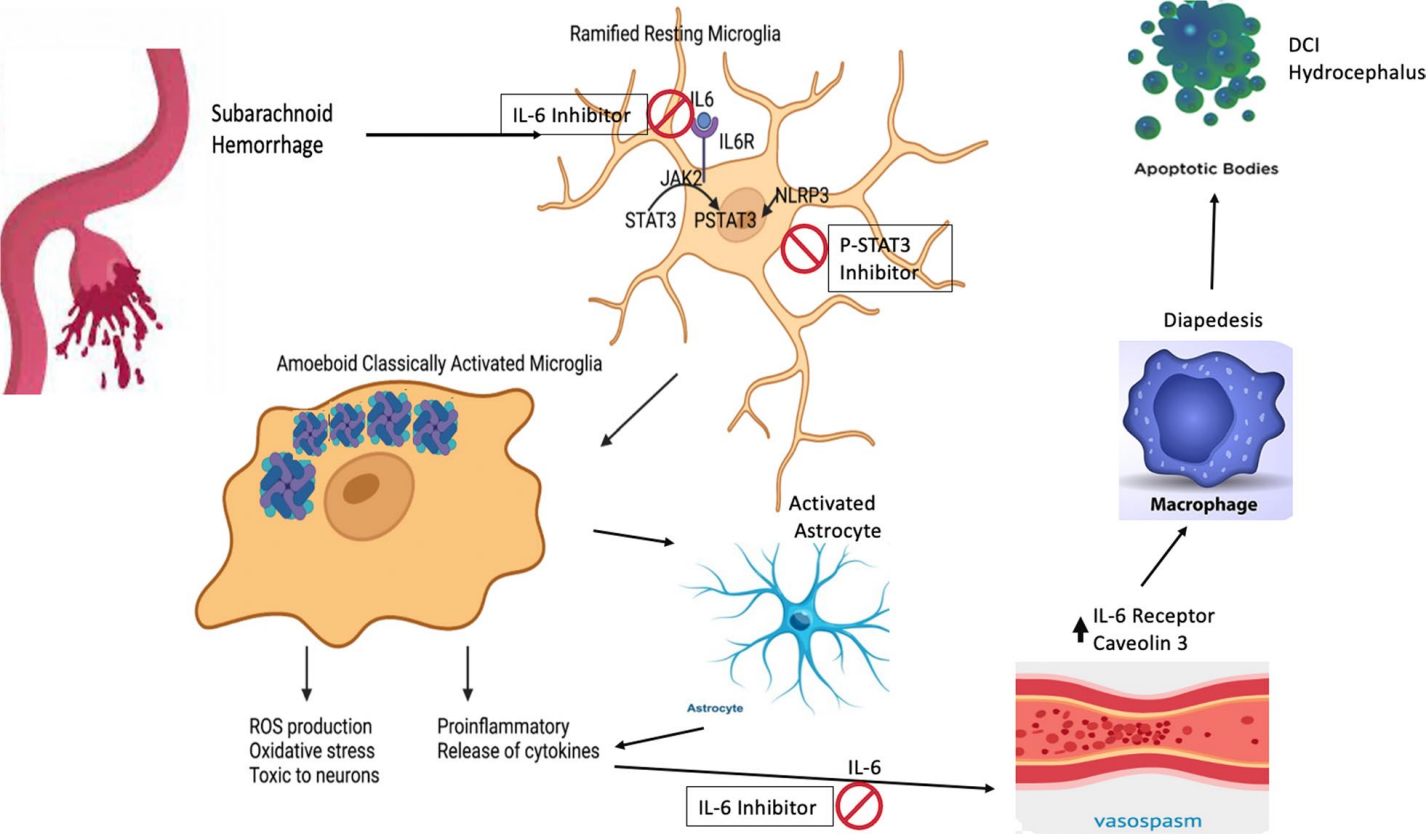

## Introduction

Subarachnoid hemorrhage (SAH) is associated with high morbidity and mortality. 70% of SAH patients develop imaging characteristics of vasospasm and 30% become clinically symptomatic [1]. An increase in interleukin-6 (IL-6) has been shown to shortly precede vasospasm in both clinical and pre-clinical studies [2]. Underlying mechanisms for why this occurs have been poorly elucidated. Emerging interest has led several groups to review the clinical and pre-clinical literature regarding IL-6’s role in vasospasm development [3]. The temporal relationship is unmistakable. The spike in IL-6 in both pre-clinical and clinical specimens occur within a 3- to 5-day window and can remain elevated for several days thereafter [4]. The few pre-clinical studies targeting IL-6 following SAH have found reduced cell death and decreased evidence of delayed cerebral ischemia (DCI) [5].

In our recent review, we postulate the important role that the IL-6 pathway plays in the downstream consequences of SAH [6]. IL-6 release has been closely tied to reactive astrocytes that are critical in blood product breakdown following SAH [7]. Once IL-6 is released, it initiates a robust perivascular inflammatory response [8]. Recent evidence shows that part of the inflammatory response involves microglia activation, and in particular a desensitized amoeboid phenotype that may have a variety of morphologies [9]. These activated microglia release cytokines such as IL-6 that contribute to peripheral macrophage diapedesis. This paper looks at some of the downstream modulation on neuroinflammation with regard to targeting IL-6. Activated microglia express toll-like receptor 4 and can initiate the STAT3 pathway, which has been linked to DCI via inflammatory storming from macrophage infiltration. The pathway initiates a pro-inflammatory positive feedback loop for IL-6, which allows ongoing activity. To mechanistically confirm the role of the IL-6 pathway, we selectively targeted p-STAT3 with a custom inhibitor. The activated pathway converts traditional senescent microglia into a pro-inflammatory state that in conjunction with macrophage diapedesis causes cortical neuronal apoptosis, micro-destruction of the blood brain barrier (BBB), and hydrocephalus [10]. In this paper, our objective was to critically investigate the mechanistic IL-6 pathway with a focus on the induction of vasospasm via the initiation of this inflammatory pathway and its downstream STAT3 cascade. Utilizing both transgenic knock outs, dual-labeled microglia/myeloid mice, a focused IL-6 inhibitor, and a custom p-STAT3 inhibitor we tease apart a contributory mechanism to vasospasm. 

## Methods

### Human samples

Human blood and cerebral spinal fluid collection from aneurysm patients was approved by University of Florida Institutional Review Board (IRB202002035). Patient data were deidentified for storage into the database. Four female patient samples were collected from ruptured cerebral aneurysm blood at time of endovascular treatment of aneurysm. 6 ml of blood was collected from microcatheter positioned adjacent to ruptured aneurysm (representative of cerebral circulation blood). 6 ml of blood was collected from sheath in the wrist or groin (radial or femoral blood representative of systemic circulation). Blood was stored on ice and immediately spun in centrifuge post-procedure for processing. Serum was collected and stored in − 80 °C freezer until use for western blot. These four patients had anterior circulation aneurysms that were coiled, Hunt and Hess score of 2 or 3 at time of presentation, and a modified Fisher score of 3 or 4. All these patients had an external ventricular drain placed as well with cerebrospinal fluid collected at time of aneurysm coiling. Patients were excluded if no external ventricular drain was placed. The cerebrospinal fluid was compared to normal controls (pseudotumor patients) who had cerebrospinal fluid collected via lumbar puncture during diagnostic workup (IRB202200182). All cerebrospinal fluid samples were stored at − 80 °C prior to processing via ELISA. Similarly, aneurysm peripheral blood was compared to control peripheral blood samples via ELISA.

### Animals

**Fig. 1:**
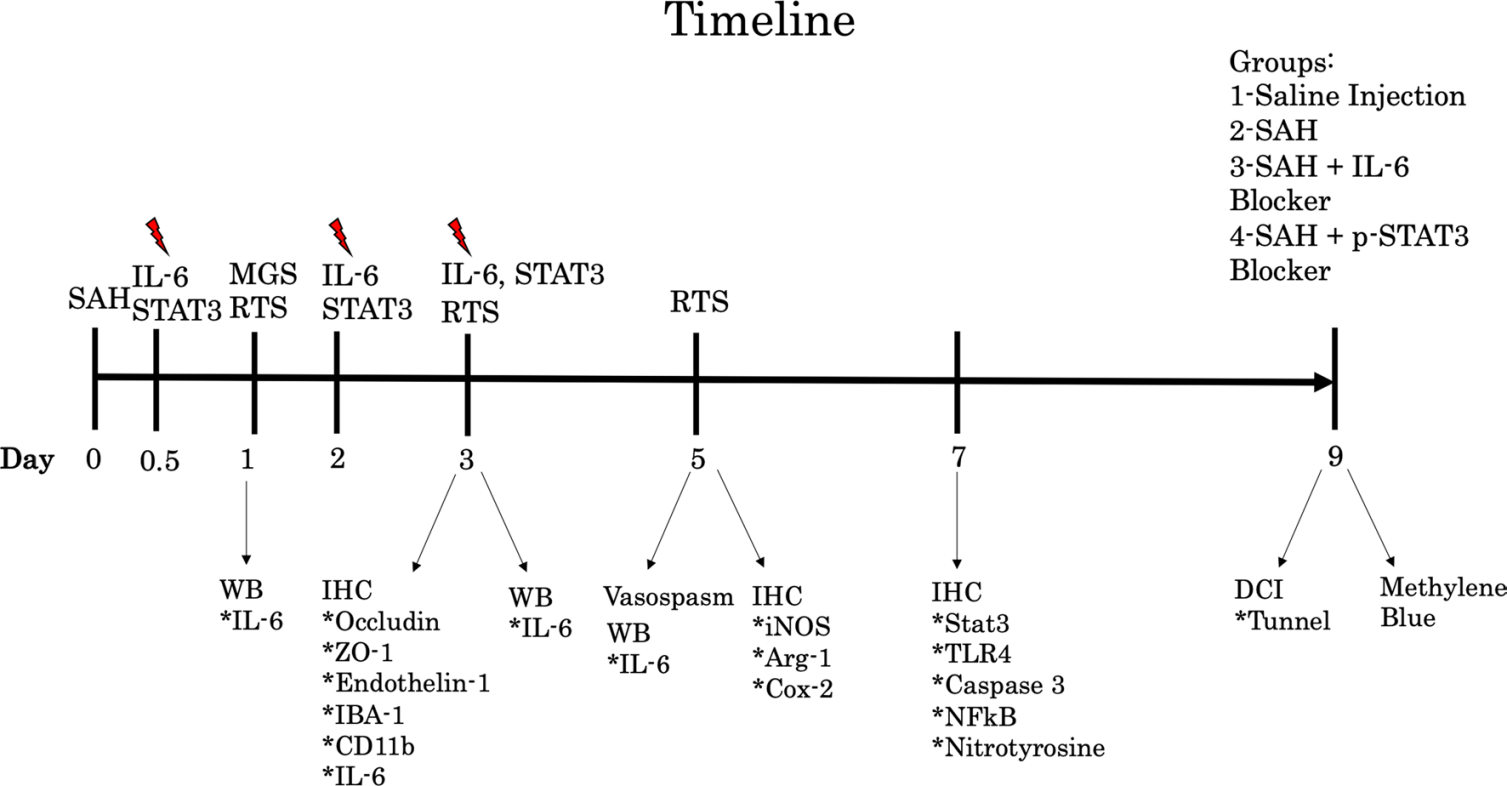
Timeline showing experimental course for assays and drug administration in wild-type cohorts. *RTS* turn test, *red lightning bolt* time of IL-6 inhibitor administration

All animal experiments were performed in accordance with the Animal Research: Reporting of In Vivo Experiments guidelines and were approved and supported by the University of Florida Institutional Animal Care and Use Committee. All animals were either C57BL/6 wild type, IL-6 knockout mice bred in C57BL/6 background, or CCR2-/CXCR1 mice graciously donated by the Harrison Laboratory. Mice were between 12- to 15-week-old females. They were housed with ad libitum food and water and 12-h light/dark cycling. All wild-type mice were received from Charles River Labs (Wilmington, MA). IL-6 knockout mice were received from The Jackson Laboratory (Bar Harbor, ME). Cages were randomly assigned to groups to make experimental groups even prior to surgical intervention. Experimental timeline is outlined in Fig. 1.

### Subarachnoid hemorrhage

The SAH model utilized modified autologous blood injection to basal cisterns adjacent to right anterior circulation [11]. Briefly, mice were anesthetized with 10 mg/kg xylazine and 100 mg/kg ketamine prior to shaving/cleaning the scalp. The tail was prepped, shaved, and cleaned. The mouse’s head was fixed to the stereotaxic frame and body temperature maintained at 37 °C using a heating pad. The scalp was incised along the sagittal suture from bregma to the nasal bone. The skin was reflected and held in place by retraction. The burr hole was positioned 5.0 mm rostral to bregma and 0.5 mm right of midline. Next, 150µL of arterial blood was drawn from the tail artery. 50µL of blood was drawn up into a Hamilton syringe. 50µL was chosen in accordance with the reported range for animal weights used in this study (19–24 g). 30µL-100µL has been reported in the literature based on a recent systematic review with the primary adjustment being according to animal weight [12]. The syringe needle was passed at a 30° caudal angle until it reached the skull base. The syringe was left in place and blood was manually injected at a rate of 10µL/min. The mouse’s respiratory rate was closely monitored. Sham procedure was identical to the above except no tail blood was collected, but instead saline injected. Once the needle was removed, the skin flap was sutured with 5-0 ethilon suture. The mice were administered buprenorphine as an analgesic and placed on a warmer until recovered from anesthesia.

### Vasospasm

Ten mice per group were utilized. Five days post-SAH, mice were deeply anesthetized with ketamine and xylazine. Cardiac catheterization was performed, and perfusion was done with 5 ml PBS followed by 15 ml of 4% paraformaldehyde. 20% India ink was dissolved in 5% gelatin and heated. 2 ml of India ink solution was perfused at a rate of 15 ml/min after the administration of paraformaldehyde. The mouse carcass was then stored at 4 °C for 6 h to allow hardening. Brains were imaged and the ipsilateral anterior cerebral and middle cerebral artery were captured. The narrowest diameter within the M1 segment was measured as a ratio against average A1 segment diameter. Mice with bifurcated or aplastic vessels were excluded. The ratio is a reliable measure of vasospasm as previously reported [11].

### Cerebral blood flow

Four mice per group were utilized. Cerebral blood flow was measured with a laser speckle perfusion imager at 48 h after SAH. The protocol was like that previously described [13]. Briefly, mice were anesthetized, scalp prepped, and skin removed to reveal skull surface. Sufficient readings were able to be achieved without bone flap removal. The speckle perfusion imager was placed ~ 10 cm above the skull and leveled to a baseline value of 100 (Pericam PSI System, PeriMed). Recordings were done for 2 min per animal. A color-coded image was displayed and converted into numerical scoring based on computer algorithm (PIMsoft, Perimed, Stockholm, Sweden). Average perfusion per animal was documented and an imaging voxel with a calculated mean of 9.7 was used to measure bilateral MCA territory perfusion. A ratio of right to left MCA perfusion was calculated for each animal to allow reliable comparisons between groups and to control for variance in baseline blood pressure differences.

### Pharmaceutical dosing

To neutralize IL-6, intraperitoneal injection of IL-6 blocking antibody (Bio X Cell, Lebanon, NH) was administered at a concentration of 10 µg/g at 12 h post-blood injection, 48 h post-blood injection, and 72 h post-blood injection. Dosing was chosen based on prior experimental efficacy with C57BL/6 mice [14]. For the knockout mice, to confirm effect of IL-6, endogenous administration of IL-6 was performed 6 h prior to blood injection and then daily until time of euthanasia. Recombinant IL-6 (Peprotech, Inc., Rocky Hill, NJ, USA) was administered at 3.2 ng/g per day based on prior protocols [15]. To confirm pathway specificity, we worked with our medicinal chemist colleague to develop a custom p-STAT3 inhibitor that selectively crosses the blood brain barrier. LLL12B was administered at 5 mg/kg dosing 1, 2, and 3 days after SAH.

### Neurologic evaluation

Eight mice per group were utilized. Mice were observed and scored based on the modified Garcia score and corner test. The modified Garcia score consists of six tests involving assessment of whisker stimulation, climbing, body proprioception, forepaw outstretching, spontaneous activity, and movement of four limbs [16]. For the corner test, two 20-cm boards were adjoined at 30° and placed on a clean testing environment. Mice were acclimatized to the apparatus for 10 min prior to SAH or saline administration. The mice were tested post-SAH as previously described [17]. Briefly, the mouse was placed directly in the middle of the two boards with nose facing the angle. The mouse entered deep into the corner and would rear to turn. The turn was recorded as either right or left. 10 total turns were recorded for each animal per day.

### Western blot and ELISA

Six mice per group were utilized for protein collection from either blood or right frontal cortex as previously described [18]. Briefly, for blood collection six mice per group were anesthetized and 2 ml of blood collected via cardiac catherization. The blood was spun at 3000 rpm for 15 min, and serum collected for protein analysis. For brain tissue collection, six mice per group were anesthetized and perfused with 10 ml of ice-cold phosphate buffered saline (PBS). Rapid decapitation with careful removal of brain and placement on brain matrix (Harvard Apparatus, Holliston, MA) was performed. The right frontal cortex was sectioned and snap-frozen in liquid nitrogen. Once frozen, it was homogenized with a Dounce homogenizer and agitated in radio-immunoprecipitation assay (RIPA) buffer for 90 min. Samples were centrifuged, supernatant collected, and protein quantified via the Bradford assay. Samples were stored in -20 °C. 35 μg of protein was separated on 4–15% Tris–glycine gels at 90 V for a total of 60 min. The protein was then transferred to nitrocellulose membranes. Membranes were blocked with 5% skim milk in Tris-buffered saline tween 20 (TBS-T) for 1 h at room temperature. The blocking solution was washed off with rinses of TBS-T and membrane probed with primary antibodies overnight at room temperature. Primary antibodies utilized were rabbit anti-mouse IL-6 28 kDa (1:1000, Abcam, Branford, CT), rat anti-mouse β-actin 42 kDa (1:5000, Cell Signaling, Danvers, MA), and rabbit anti-mouse p-STAT3 88 kDa (1:1000, Cell Signaling, Danvers, MA). The membranes were then washed with TBS-T and incubated with HRP-conjugated secondary antibody (1:2000, Abcam, Branford, CT) for 1 h at room temperature. Membranes were washed and incubated in luminol-peroxide solution (Santa Cruz Biotechnology, Dallas, TX) for 1 min and taken to the dark room. Films were developed, and densitometry performed with ImageJ software. Band densities for protein of interest were normalized to β-actin. ELISA was done according to manufacturer instructions per kit (Abcam, Waltham, MA).

### Immunohistochemistry

Six mice per group were utilized. Mice were anesthetized and cardiac perfused with PBS at a rate of 5 ml/min. Brains were removed, placed in plastic cassette with OCT, and submerged in − 65 °C isopentane. The blocks were stored at − 80 °C. On day of sectioning, the OCT blocks were mounted for the Leica CM3050S cryostat (Leica Microsystems) and sliced at a thickness of 14 μm. Slices were collected on a mounted glass slide. The staining protocol was similar to that previously published [19]. After initial serial washes, brain slices were incubated overnight with primary antibodies according to manufacture recommended dilutions. Antibodies used: Endothelin-1, ZO-1, Occludin, GFAP, p-STAT3, Arg1, Cox2, NFΚB, and caspase 3 (Cell Signaling, Danvers, MA), IL-6R, IL-6, iNOS, Caveolin 3 (Abcam, Branford, CT), IBA-1, CD11B (Invitrogen, Waltham, MA), nitrotyrosine (Millipore Sigma, Burlington, MA), and TLR-4 (Proteintech, Rosemont, IL). The slides were then rinsed, and Alexa Fluor secondary antibodies were applied to the slides for 3 h. The slide was rinsed and Vectashield 4’,6-diamidino-2-phenylindole (DAPI) nuclear counterstain was applied (Vector, Burlingame, CA).

Right frontal and periventricular regions were examined. Ten slides per animal were prepared. Olympus IX71 fluorescent microscope (Olympus America, Center Valley, PA) was used. For fluorescent assays, total corrected cell fluorescent protocol was utilized as previously described [18]. For cellular morphology assays, total number of cells per high power field were compared as previously described by blinded observer [20]. This method was utilized for NeuN-stained slides with Tunel assay according to manufacturer instructions (Abcam, Cambridge, MA). For co-localization assays, the Just Another Co-localization Plugin from ImageJ was utilized with Costes correction [21]. The vessel disruption percentage was calculated as percentage of luminal irregularity by methods previously described [22]. Ventricle post-mortem size assessment was done with methylene blue staining according to methods previously described [23]. Clarity analysis was done according to standard protocol [24].

### Statistical analysis

Western blot, Tunel, immunohistochemistry, vasospasm, and behavioral assays were evaluated with one-way ANOVA with Tukey post hoc comparison to determine differences between groups. For comparison between two groups, a Student’s *t*-test was performed. Power calculations were completed for vasospasm with *α* = 0.05, *β* = 0.2, and minimum difference expected between groups of 20%. Overlap coefficient was determined with ImageJ software and the Just Another Co-localization Plug In. GraphPad Prismv8.0 (GraphPad Software, San Diego, CA) was utilized for statistical analysis with *p* < 0.05 considered significant.

## Results

### IL-6 peaks on day 3 and has periventricular and perivascular release

**Fig. 2:**
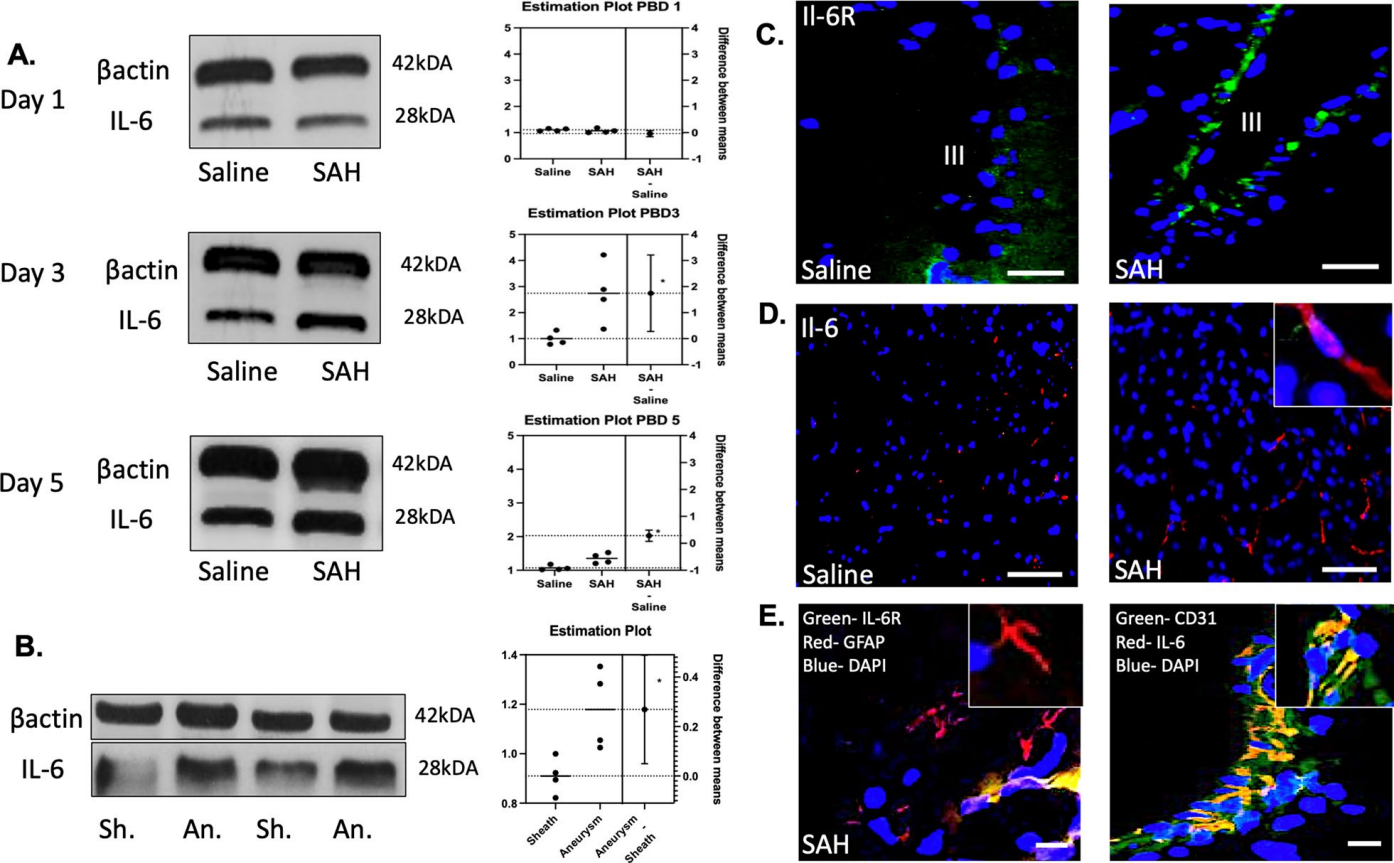
IL-6 measurements in both peripheral blood and brain. A peak in IL-6 was seen on post-SAH day 3 and sustained on post-SAH day 5 (*p* < 0.05) (**A**). IL-6 was also increased in aneurysmal blood versus sheath blood in human samples (*p* < 0.05) (**B**). IL-6 receptor increase was seen in perivascular distribution on post-bleed day 3 (**C**). IL-6 release was perivascular in distribution (**D**). Co-localization revealed surrounding reactive astrocytes and IL-6 increased expression with endothelial markers (**E**). White bar panel **B** = 60 μm, **C** = 50 μm, panel **D** = 30 μm

IL-6 was measured in peripheral blood on Day 1, 3, and 5 following SAH. No significant difference was seen on day 1 post-SAH with a mean difference of 0.04 between saline and SAH (*t* = 0.79, *p* = 0.46). A significant increase was seen on day 3 post-SAH with a mean difference 1.75 between saline vs. SAH (*t* = 2.91, *p* = 0.027). A continued peak was seen on day 5 post-SAH with a mean difference 0.28 between saline vs. SAH (*t* = 3.33, *p* = 0.016) (Fig. 2A). IL-6 was increased in perianeurysmal blood compared to sheath blood for human samples at time of aneurysm treatment with mean difference 0.269 (*t* = 3.001, *p* = 0.024) (Fig. 2B). IL-6 receptor upregulation within the brain was seen in a periventricular distribution (Fig. 2C). IL-6 measurements within the brain were upregulated near the periventricular space (Fig. 2D). When co-localized with astrocytic marker GFAP and endothelial marker CD31, it was noted that the IL-6 expression was within the skull base microvasculature adjacent to astrocyte podocytes (Fig. 2E).

### IL-6 increases in CSF compared to control with novel target for STAT3 modulation

**Fig. 3:**
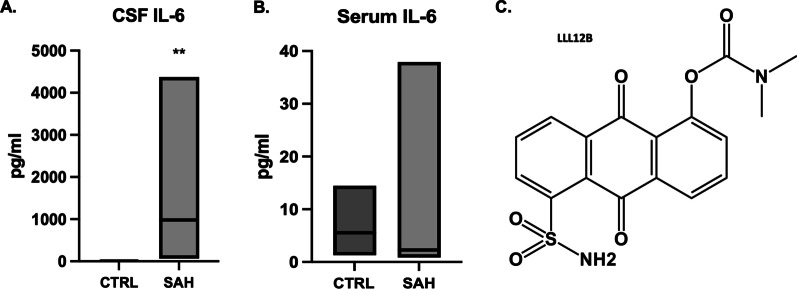
IL-6 was significantly increased in CSF of SAH patients compared to control (*p* < 0.01) (**A**). No significant change was observed between groups for peripheral serum samples (*p* = 0.55) (**B**). A selective small molecule inhibitor to target the downstream cascade of IL-6 via p-STAT3 inhibition was synthesized (**C**)

CSF was compared between SAH patients and healthy controls. Average pg/ml of IL-6 in control CSF was 5 compared to 1686 for SAH patient CSF (*t* = 3.29, *p* < 0.01) (Fig. 3A). No significant difference was seen in IL-6 levels between peripheral IV serum samples between groups. Average pg/ml of IL-6 in control peripheral serum was 5 compared to 8 for the SAH patients (*t* = 0.61, *p* = 0.55) (Fig. 3B). The CNS-specific surge in IL-6 prompted the development of a focused small molecule inhibitor LLL12b that could cross the BBB and selectively target inflammation and oxidative stress. Chemical formulation is outlined in Fig. 3C.

### IL-6 blockade mitigates blood brain barrier disruption and microglia activation

**Fig. 4:**
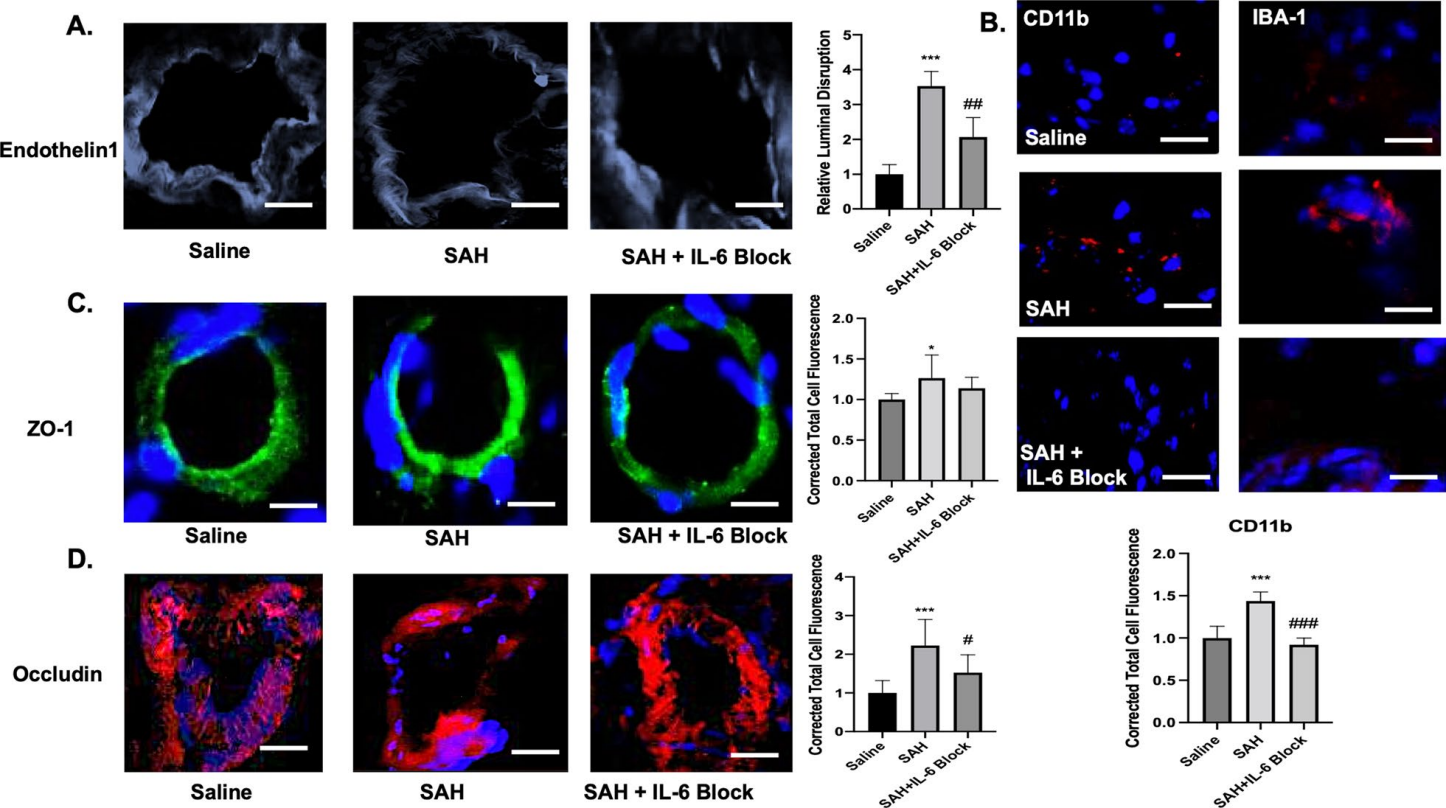
Blood brain barrier disruption and perivascular microglia recruitment was assessed on post-bleed day 3. A significant increase in luminal disruption was seen following SAH (*p* < 0.001) that was mitigated by IL-6 blockade (*p* < 0.01) (**A**). Perivascular microglia recruitment was observed as measured with IBA-1 and CD11b following SAH (*p* < 0.001) that was significantly reduced with IL-6 blockade (*p* < 0.001) (**B**). ZO-1 was significantly increased following SAH (**C**). Occludin was significantly increased following SAH (*p* < 0.001) but reduced with IL-6 blockade (*p* < 0.05) (**D**). * = significance compared to saline, ^#^significance compared to SAH. White bar = 50 μm

Post-SAH day 3 endothelin-1 staining was done to look at luminal irregularity in microvasculature of right MCA region. A significant difference was seen between groups (*F*(2,9) = 34.48, *p* < 0.001). The mean difference was 2.53 between saline and SAH (*q* = 11.7, *p* < 0.001). This effect was mitigated with IL-6 blockade with mean difference 1.467 between SAH vs. SAH + IL-6 block (*q* = 6.77, *p* = 0.003) (Fig. 4A). Microglia recruitment to the perivascular space was significantly different between groups as measured with CD11b and IBA-1 (*F*(2,27) = 62.79, *p* < 0.001). A mean difference of 0.44 was seen between saline vs. SAH (*q* = 12.47, *p* < 0.001). IL-6 blockade reduced the microglia recruitment with mean difference 0.52 between SAH vs. SAH + IL-6 block (*q* = 14.7, *p* < 0.001) (Fig. 4B). A significant difference was seen between groups for ZO-1 (*F*(2,27) = 4.96, *p* = 0.14). A mean difference of 0.26 was seen between saline vs. SAH (*q* = 4.45, *p* = 0.01). No significant difference was seen with IL-6 blockade (Fig. 4C). A significant difference was seen between groups for occludin (IL-6 blockade provided significant benefit in preserving occludin (*F*(2,26) = 13.75, *p* < 0.001). A mean difference of 1.23 was seen between saline vs. SAH (*q* = 7.37, *p* < 0.001). IL-6 blockade prevented occludin dysregulation with mean difference 0.7 between SAH vs. SAH + IL-6 block (*q* = 4.32, *p* = 0.014) (Fig. 4D).

### IL-6 knockout protects against vasospasm, secondary cascades, and reduction of cerebral blood flow following subarachnoid hemorrhage

**Fig. 5:**
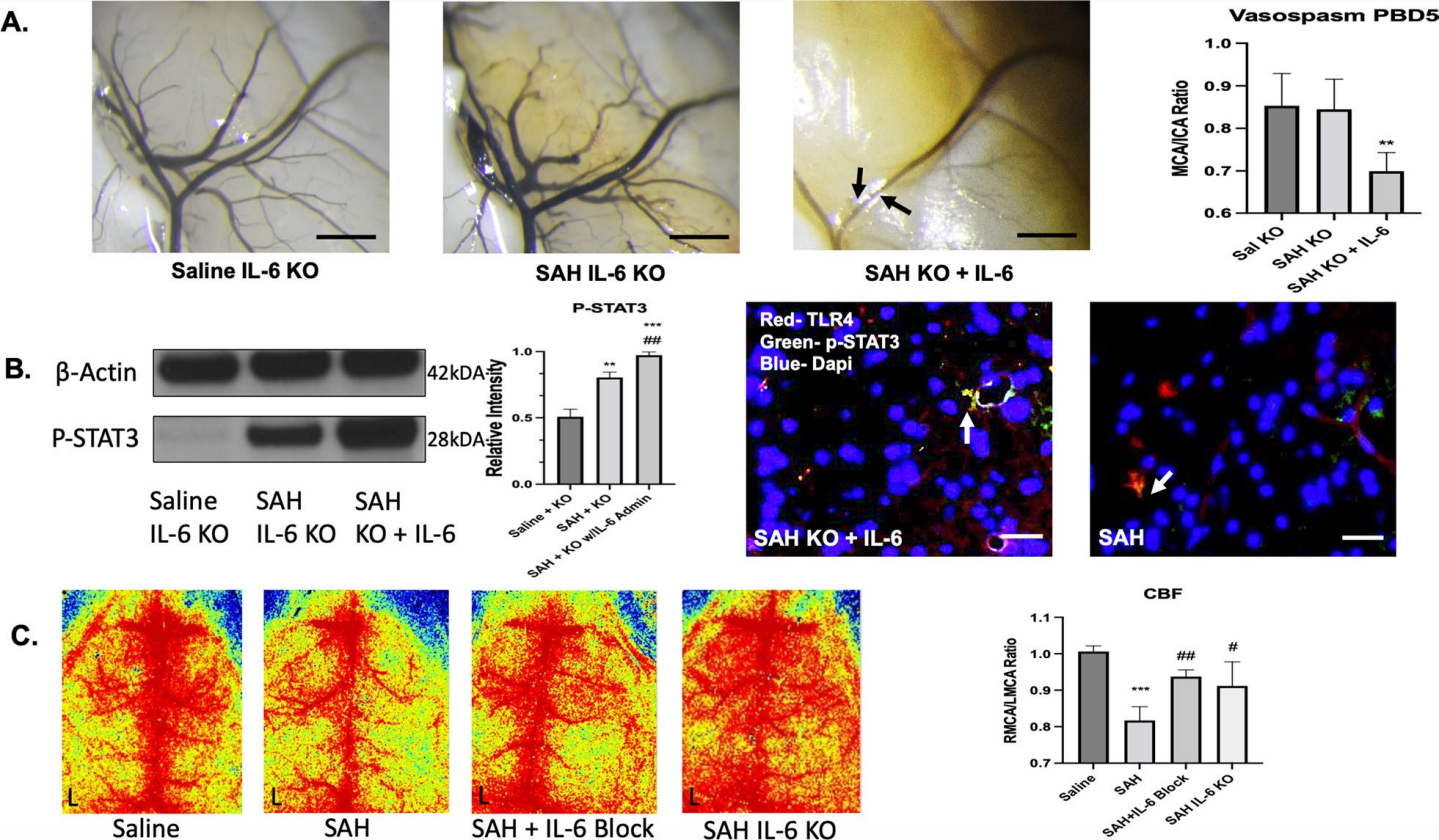
IL-6 knockout is protective against decreased cerebral blood flow, vasospasm, and secondary cascades. On post-bleed day 5, no significant increase in vasospasm was seen in the SAH IL-6 KO group. When IL-6 was administered to the knockout mice in context of SAH, vasospasm developed (*p* < 0.01) (**A**). IL-6 administration to the SAH KO mice significantly increased p-STAT3 compared to saline (*p* < 0.001) and to SAH IL-6 KO without administration (*p* < 0.01). p-STAT3 and TLR4 co-localization was similar between the SAH IL-6 KO mice with IL-6 administration and SAH wild-type mice (**B**). A significant decrease in RMCA/LMCA ratio cerebral blood flow was seen 48 h after SAH *p* < 0.001. This was partially mitigated by IL-6 blockade (p < 0.01) and IL-6 KO (*p* < 0.05) (**C**). Black scale bare = 100 μm. White scale bar = 50 μm. *comparison to saline. ^#^**b** = comparison to SAH + KO. ^#^**c** = comparison to SAH

A significant difference between groups was seen for knockout mice on post-SAH day 5 (*F*(2,12) = 8.85, *p* = 0.004). IL-6 knockout mice displayed no evidence of vasospasm compared to saline. When systemic IL-6 was given to the knockout mice immediately prior and after SAH, vasospasm resulted compared to saline with a mean difference 0.15 (*q* = 5.291, *p* = 0.007) (Fig. 5A). To investigate downstream mechanisms, p-STAT3 western blot was utilized and revealed a significant difference between groups (F(2,9) = 118.5, p < 0.001). Post hoc analysis revealed mean difference 0.3 between saline vs. SAH KO (*q* = 7.77, *p* = 0.008), mean difference 0.47 between saline vs. SAH KO with endogenous IL-6 administration (*q* = 21.5, *p* < 0.001), and mean difference 0.169 between SAH KO and SAH KO with endogenous IL-6 administration (*q* = 13.73, *p* = 0.002). p-STAT3 was co-localized with TLR4 in a similar perivascular distribution for the SAH KO with endogenous IL-6 administration compared to SAH wild type (Fig. 5B). A significant difference in blood flow was seen between groups (*F*(3,12) = 15.42, *p* < 0.001). A reduction in right MCA cerebral blood flow was seen for SAH mice vs. saline with mean difference 0.19 (*q* = 9.49, *p* < 0.001). This was mitigated by IL-6 blockade vs. SAH with mean difference 0.12 (*q* = 6.04, *p* = 0.005), and IL-6 KO vs. SAH with mean difference 0.09 (*q* = 4.75, *p* = 0.026) (Fig. 5C).

### Peripheral macrophages enter post-SAH day 5; IL-6 blockade blunts caveolin increase

**Fig. 6:**
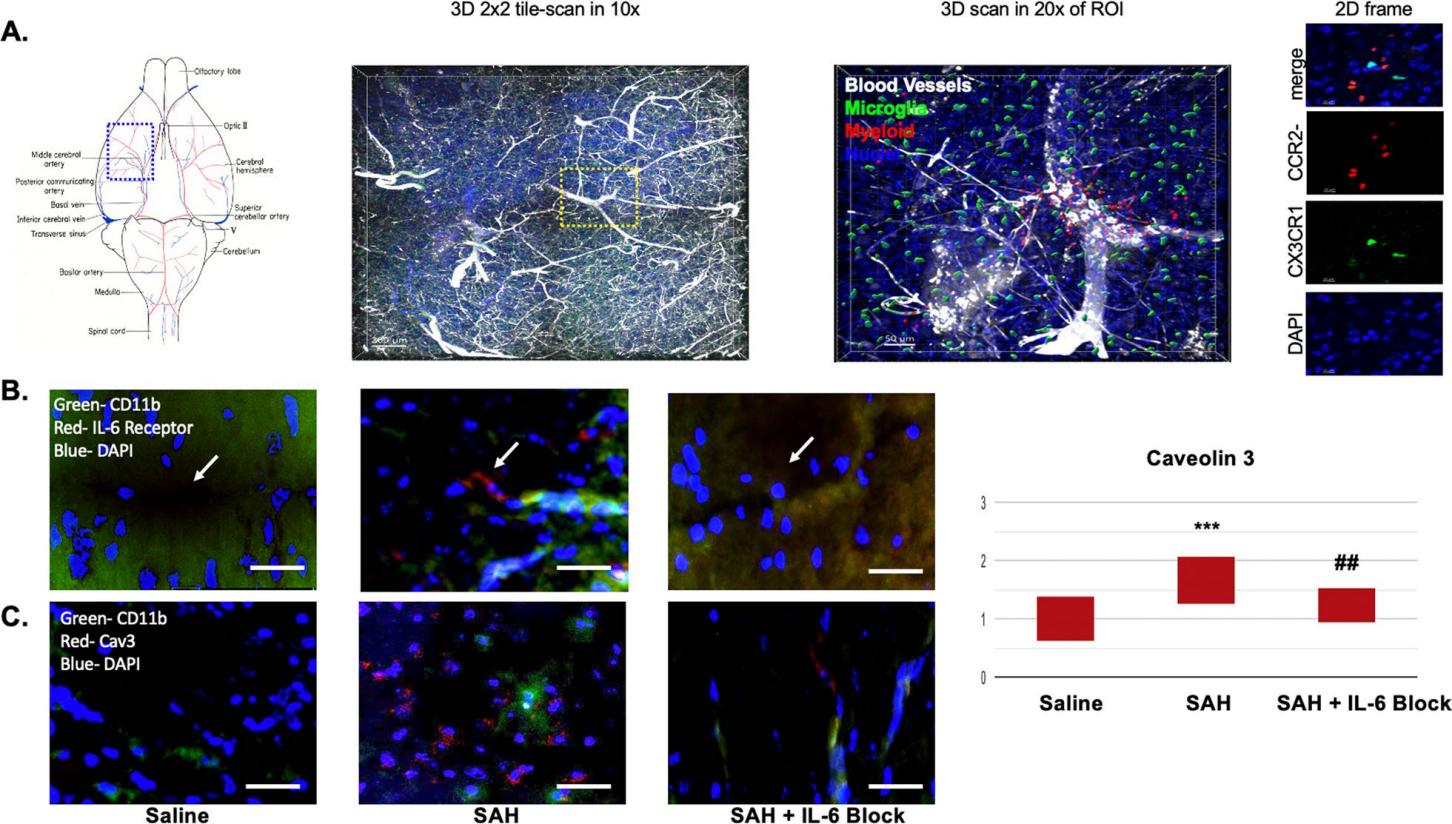
On post-bleed day 5, a significant infiltration of myeloid derived cells was noted in the region of vasospasm (**A**). Increased IL-6 receptors were seen in microvascular beds adjacent to the region of vasospasm and microglia activation (**B**). A significant increase in Caveolin 3 was seen following SAH (*p* < 0.001). IL-6 blockade provided benefit against the caveolin increase thereby helping reduce diapedesis (*p* < 0.01) (**C**). White scale bar = 50 μm. * = comparison to saline. ^#^ = comparison to SAH. Arrows point to microvascular region in each panel

In regions of vasospasm, it was noted that peripheral myeloid cells (macrophages) infiltrated adjacent to regions of microglia activation (Fig. 6A). This was seen along the MCA distribution internal to where the SAH blood was noted. The myeloid infiltration was not seen in regions that were not associated with vasospasm. A significant increase in IL-6 receptors was associated in microvasculature surrounding the region of vasospasm and adjacent to activated microglia (Fig. 6B). These same regions had a significant increase in caveolin 3 (Fig. 6C). A significant difference between groups was noted (*F*(2,57) = 15.64, *p* < 0.001). Post hoc analysis revealed a significant difference between saline (mean 1) and SAH (mean 1.65) (*p* < 0.001). IL-6 blockade provided protective benefit against SAH (mean 1.23) (*p* < 0.01).

### Pharmacologic IL-6 blockade prevents vasospasm and improves neurologic performance

**Fig. 7:**
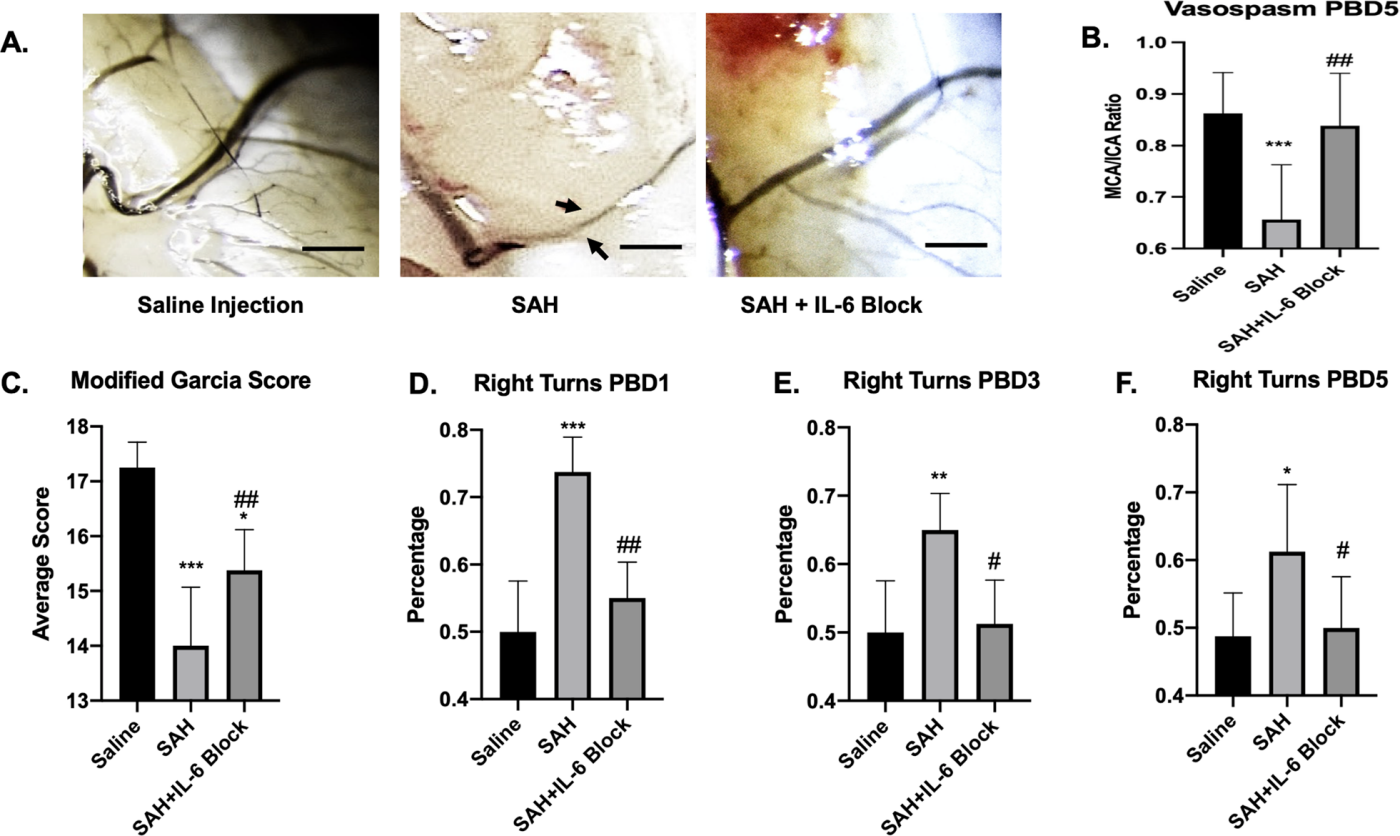
IL-6 blockade prevents vasospasm and improves neurologic scores. Schematic of SAH model set-up (**A**). Vasospasm measurements with arrows showing areas of vasospasm (**B**). A significant decrease in MCA/ICA ratio seen after SAH (*p* < 0.001). This was mitigated by IL-6 blockade (*p* < 0.01) (**C**). Mice with SAH had worse modified Garcia scale scores (*p* < 0.001), but improvement was seen with IL-6 blockade (*p* < 0.01) (**D**). Percentage of right turns (indicating impaired left motor functioning) was seen on post-SAH day 1 (*p* < 0.001) with improvement with IL-6 blockade (p < 0.01) (**E**). This effect was maintained on post-SAH day 3 (*p* < 0.01) with continued IL-6 blockade benefit (*p* < 0.05) (**F**). By post-SAH 5, the SAH mice were returning towards baseline but still had significant difference (*p* < 0.05) with benefit from IL-6 blockade (*p* < 0.05) (**G**). Black scale bar = 100 μm. * = comparison to saline, # = comparison to SAH

Significant difference between groups was seen for vasospasm post-SAH day 5 (*F*(2,24) = 11.44, *p* < 0.001). Vasospasm was noted between saline versus SAH with mean difference 0.21 (*q* = 6.28, *p* < 0.001). IL-6 blockade mitigated this effect with mean difference versus SAH 0.19 (*q* = 5.39, *p* = 0.003) (Fig. 7A, B). A significant difference was also noted between groups for modified Garcia scores (*F*(2,21) = 33.43, *p* < 0.001). SAH mice has worse scores compared to saline (14 vs. 17.25 with *q* = 11.52, *p* < 0.001) IL-6 blockade provided some protective benefit versus SAH (mean difference 1.38, *p* = 0.007) (Fig. 7C). A significant difference between groups was seen for the right turn test post-SAH day 1 (*F*(2,21) = 33.44, *p* < 0.001), post-SAH day 3 (*F*(2,21) = 13.11, *p* < 0.001, and post-SAH day 5 (*F*2,21) = 5.79, *p* < 0.01). SAH mice spent 74% turning to right (indicating left sided deficits) on day 1 compared to 50% for saline and 55% for IL-6 blockade (saline vs. SAH *q* = 10.97, *p* < 0.001; SAH vs. SAH + IL-6 blockade *q* = 8.66, *p* < 0.01). On day 3 SAH mice spent 65% turning to right compared to 50% for saline and 51% for IL-6 blockade (saline vs. SAH *q* = 6.52, *p* < 0.01; SAH vs. SAH + IL-6 blockade *q* = 5.9, *p* < 0.05). On day 5, SAH mice spent 61% turning right compared to 48% saline and 50% IL-6 blockade (saline vs. SAH *q* = 4.37, *p* = 0.015; SAH vs. SAH + IL-6 blockade *q* = 3.93, *p* = 0.03) (Fig. 7D–F).

### Subarachnoid hemorrhage induces microglia alteration

**Fig. 8:**
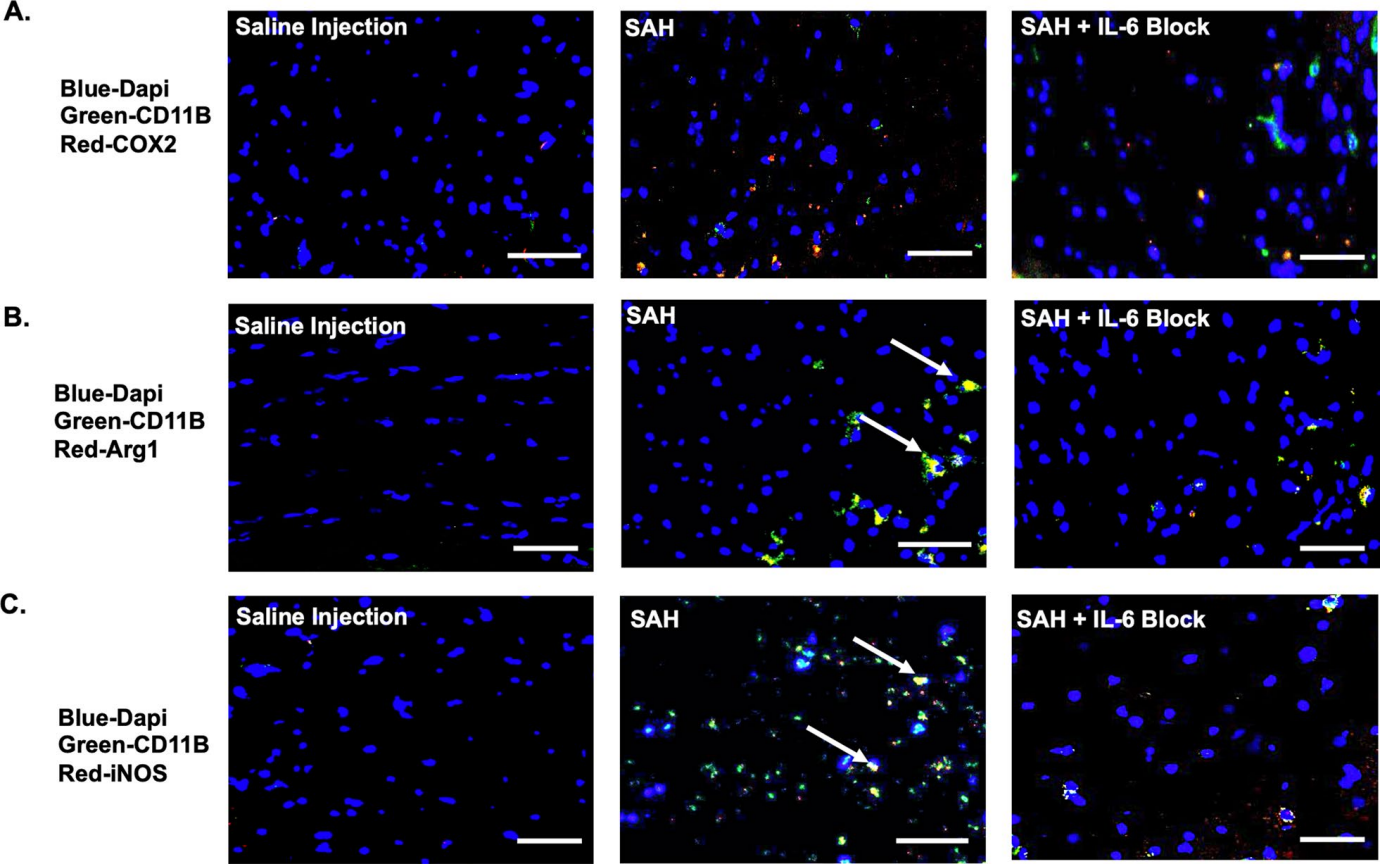
Microglia activation was seen 5 days following SAH with IL-6 blockade blunting effect. CD31 and COX2 overlap coefficients for saline r = 0.78, SAH r = 0.65, and SAH + IL-6 blockade r = 0.81 (**A**). CD31 and Arg1 overlap coefficients for saline r = 0.77, SAH r = 0.96, and SAH + IL-6 blockade r = 0.88 (**B**). CD31 and iNOS overlap coefficients for saline r = 0.86, SAH r = 0.94, and SAH + IL-6 blockade r = 0.66 (**C**). Arrows indicate strong co-localization. Scale bar = 50 μm

On PBD5, SAH induced microglia phenotype switching. CD11b was co-localized with COX2, Arg1, and iNOS. Overlap coefficients with CD11b and COX2 for saline r = 0.78 (k1 0.73, k2 1.24), SAH r = 0.65 (k1 0.55, k2 1.45), and SAH + IL-6 blockade r = 0.81 (k1 0.76, k2 1.19) (Fig. 8A). Overlap coefficients with CD11b and Arg1 for saline r = 0.77 (k1 0.75, k2 1.28), SAH r = 0.96 (k1 0.93, k2 1.055), and SAH + IL-6 blockade r = 0.96 (k1 0.81, k2 1.21) (Fig. 8B). Overlap coefficients with CD11b and iNOS for saline r = 0.86 (k1 0.84, k2 1.078), SAH r = 0.94 (k1 0.85, k2 1.147), and SAH + IL-6 blockade r = 0.66 (k1 0.63, k2 1.6) (Fig. 8C).

### Subarachnoid hemorrhage initiates downstream cascades that are alleviated by IL-6 blockade

**Fig. 9:**
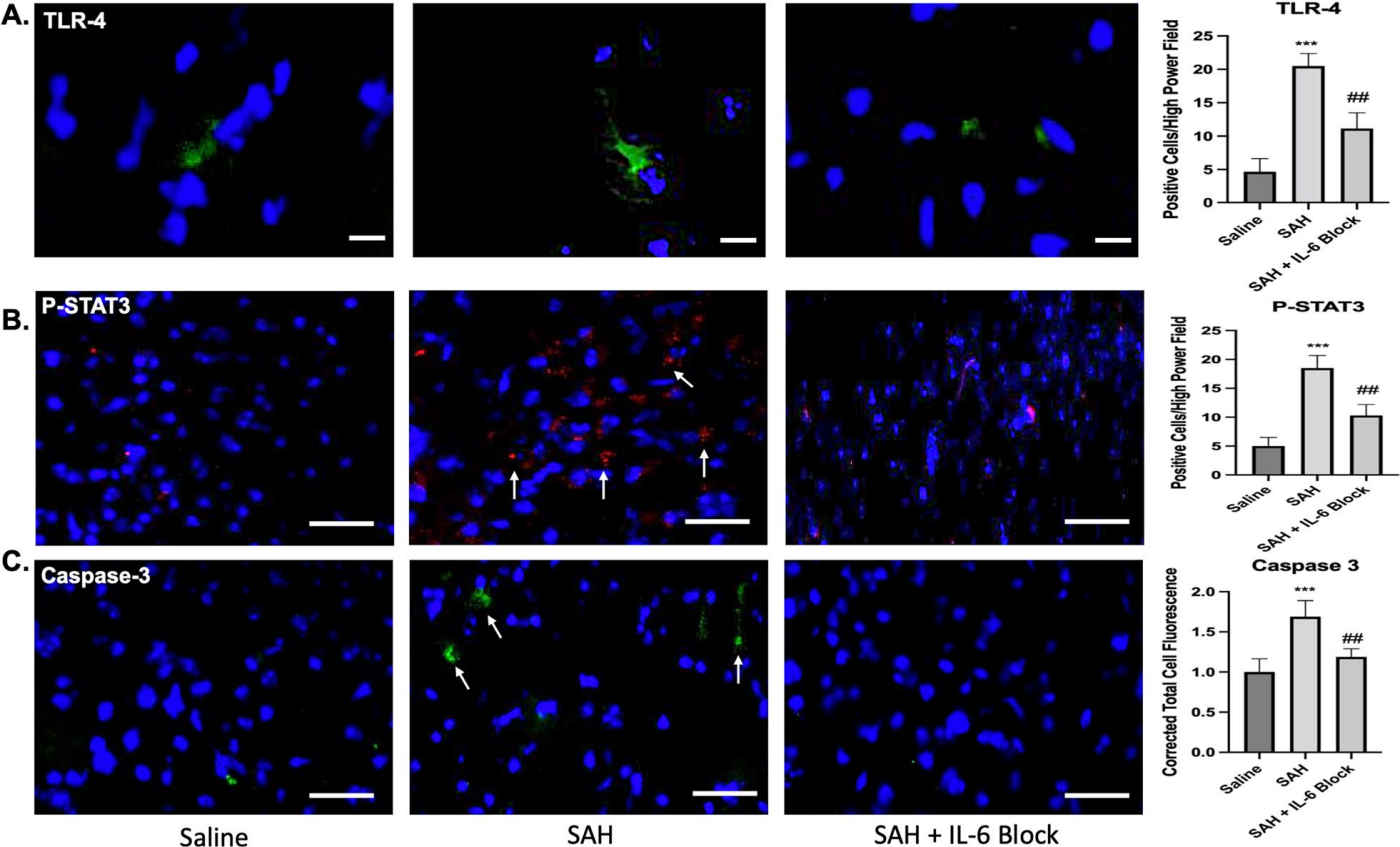
Downstream cascades were upregulated following SAH but were reduced with IL-6 blockade on post-SAH day 7. A significant increase was seen in TLR4 following SAH (*p* < 0.001), which was reduced with IL-6 blockade (*p* < 0.01) (**A**). A significant increase was seen in p-STAT3 following SAH (*p* < 0.001), with reduction by IL-6 blockade (*p* < 0.01) (**B**). A significant increase in Caspase 3 was seen following SAH (*p* < 0.001), with reduction by IL-6 blockade (*p* < 0.01) (**C**). Arrows indicate positive cells. * = comparison to saline. # = comparison to SAH. Scale bar **A** = 30 μm. Scale bar **B** and **C** = 50 μm

Toll-like receptor 4 (TLR4) expression was significantly different between groups 7 days following SAH (*F*(2,15) 89.54, *p* < 0.001). Post hoc comparison showed a mean difference of 15.83 for SAH versus saline (*q* = 18.83, *p* < 0.001). This effect was significantly reduced with IL-6 blockade versus SAH with mean difference 9.333 (*q* = 11.1, *p* < 0.01) (Fig. 9A). P-STAT3 was also significantly different between groups (*F*(2,27) 132, *p* < 0.001). A significant mean difference of 13.5 was seen between SAH versus saline (*q* = 22.81, *p* < 0.001). IL-6 blockade significantly reduced this effect with mean difference 8.2 versus SAH (*q* = 13.85, *p* < 0.01) (Fig. 9B). Caspase 3 was significantly different between groups following SAH (*F*(2,27) 49.45, *p* < 0.001). A mean difference of 0.6898 was seen between SAH versus saline (*q* = 13.61 *p* < 0.001). IL-6 blockade mitigated the increased caspase 3 activation with mean difference 0.5 versus SAH (*q* = 9.86, *p* < 0.01) (Fig. 9C).

### Selective p-STAT3 inhibition shows protective benefit of targeting IL-6 pathway

**Fig. 10:**
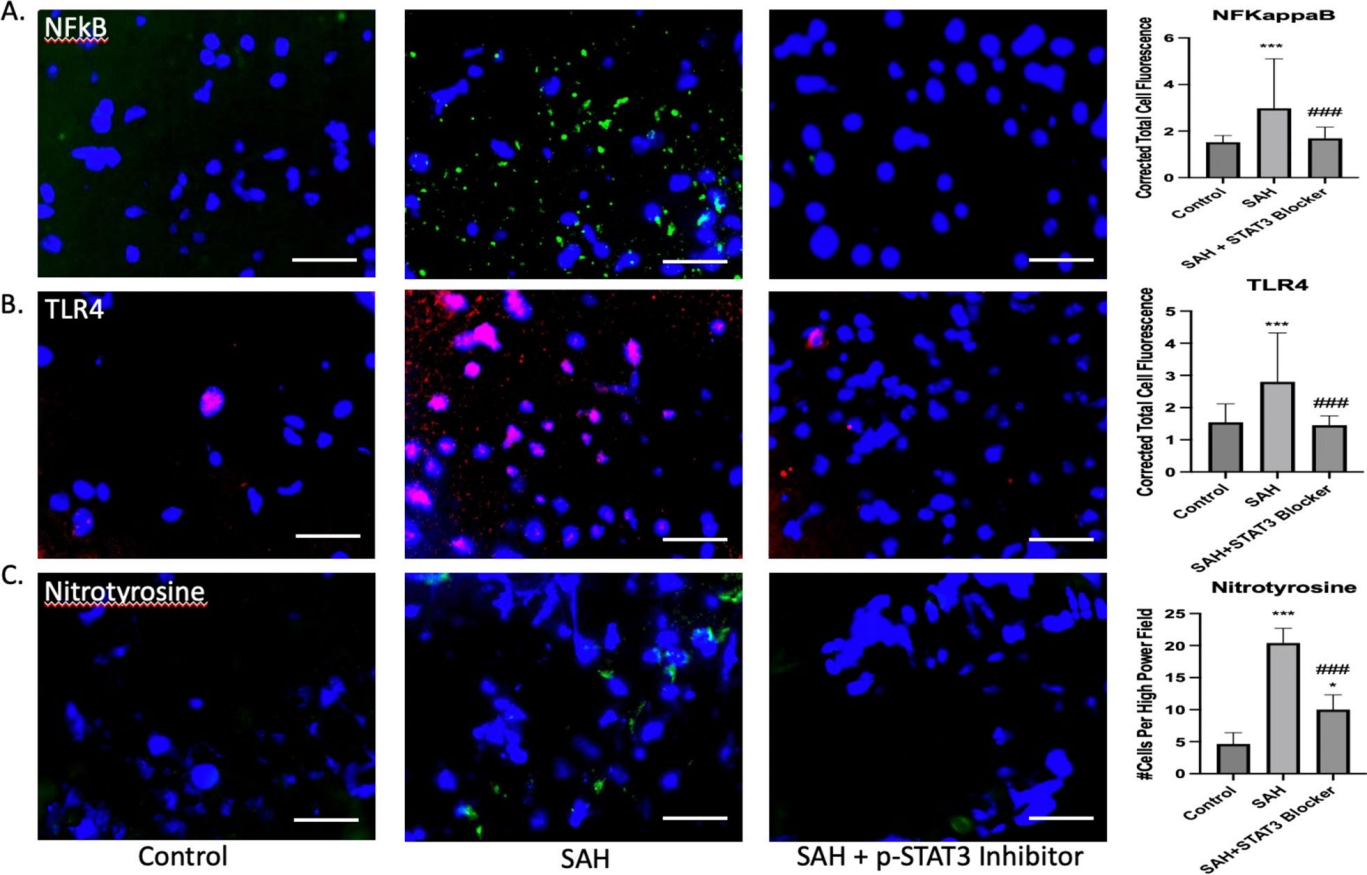
Downstream cascades were upregulated following SAH, but were reduced with p-STAT3 blockade on post-SAH day 7. A significant increase was seen in NFΚB following SAH (*p* < 0.001), which was reduced with p-STAT3 blockade (*p* < 0.001) (**A**). A significant increase was seen in TLR4 following SAH (*p* < 0.001), with reduction by p-STAT3 blockade (*p* < 0.001) (**B**). A significant increase in nitrotyrosine was seen following SAH (p < 0.001), with reduction by p-STAT3 blockade (*p* < 0.001), but not complete restoration to control levels (*p* < 0.05) (**C**). *comparison to saline. ^#^comparison to SAH. Scale bar = 40 μm

NFΚB expression was significantly different between groups 7 days following SAH (*F*(2,147), 19.54, *p* < 0.001). Post hoc comparison showed a mean difference of 1.45 for SAH versus saline (*q* = 8.07, *p* < 0.001). This effect was significantly reduced with p-STAT3 blockade versus SAH with mean difference 1.29 (*q* = 7.17, *p* < 0.001) (Fig. 10A). Toll-like receptor 4 (TLR4) expression was significantly different between groups 7 days following SAH (*F*(2,147) 31.4, p < 0.001). Post hoc comparison showed a mean difference of 1.26 for SAH versus saline (*q* = 9.36, *p* < 0.001). This effect was significantly reduced with p-STAT3 blockade versus SAH with mean difference 1.35 (*q* = 10.02, *p* < 0.01) (Fig. 10B). Nitrotyrosine was significantly different between groups following SAH (F(2,57) 281.9, *p* < 0.001). A mean difference of 15.75 was seen between SAH versus saline (*q* = 33.02, *p* < 0.001). P-STAT3 blockade mitigated the increased nitrotyrosine activity with mean difference 10.4 versus SAH (*q* = 21.8, *p* < 0.001), but did not completely abolish it compared to control (*p* < 0.05) (Fig. 10C).

### Targeting IL-6 provided protection against ventriculomegaly and cell death

**Fig. 11:**
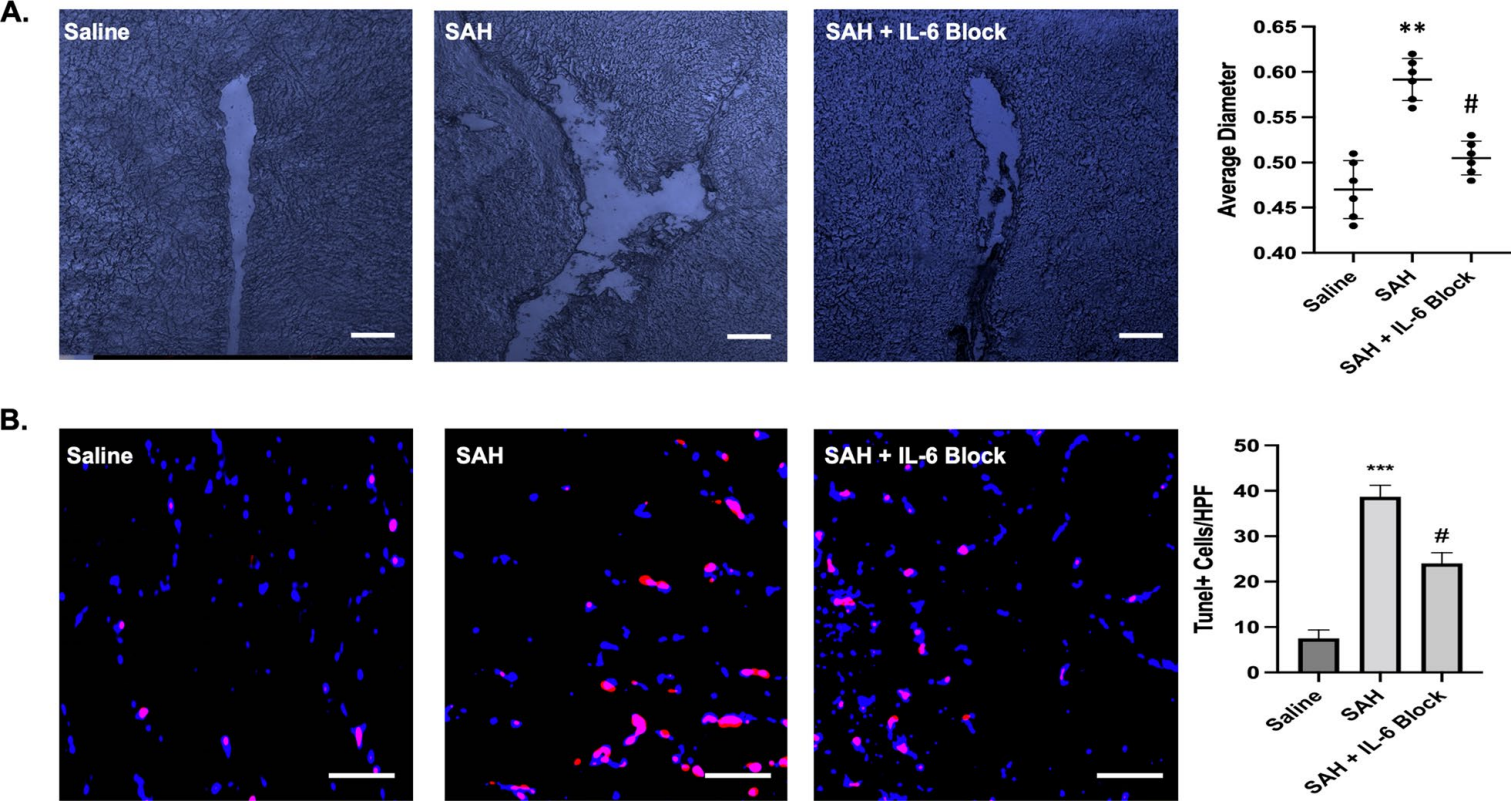
SAH caused increased ventricular dilation and cell death on post-bleed day 9, which were significantly reduced with IL-6 blockade. A significant increase in third ventricular diameter was seen on post-bleed day 9 (*p* < 0.01), with reduction back towards control levels with IL-6 blockade (*p* < 0.05) (**A**). Tunel staining was utilized to measure neuronal cell death with increases after SAH (*p* < 0.001) and reduction with IL-6 blockade (*p* < 0.05). * = comparison to saline. # = comparison to SAH. Scale bar **A** = 70 μm. Scale bar **B** = 50 μm

SAH furthermore induced increased 3^rd^ ventricle diameter on day 9 with a significant difference between groups (*F*(2, 15) = 36.65, *p* < 0.001). A mean difference of 0.12 was seen between saline versus SAH (*q* = 11.76, *p* < 0.01). IL-6 blockade prevented the ventriculomegaly consistent with hydrocephalus when compared to SAH with mean difference 0.09 (*q* = 8.38, *p* < 0.05) (Fig. 11A). SAH also induced cell death as measured with Tunel staining with a significant difference between groups (*F*(2,15) = 277.6, *p* < 0.001). A mean difference between saline versus SAH was 31.17 (*q* = 33.3, *p* < 0.001). This cell death was drastically reduced with IL-6 blockade with mean difference 14.67 (*q* = 15.67, *p* < 0.05) (Fig. 11B).

## Discussion

IL-6 has been shown clinically to peak on day 3 following SAH and is a reliable marker for vasospasm [25]. Despite strong evidence of correlation, limited work has been done regarding mechanistic contribution [26]. In this paper, we sought to investigate the mechanistic contribution following SAH through use of IL-6 blockade, IL-6 systemic KO, and STAT3 inhibition. A peak of IL-6 was seen on post-SAH day 3 and upon further assessment distribution within the brain was both periventricular and perivascular. Likewise, the human aneurysmal blood had significant increase in IL-6 compared to sheath blood. A significant increase in CSF IL-6 was seen compared to controls correlating to clinical relevance. Other groups have shown that reactive astrocytes and microglia release IL-6 at the endothelial interface following neural injury [27]. We also found a release at the endothelial interface adjacent to reactive astrocytes. This was associated with an increase in Caveolin 3. Caveolin 3 prepares the BBB for diapedesis of peripheral macrophages. Baduat and colleagues propose a complex interplay between astrocytes and microglia to facilitate this process [28]. It has been postulated that the release of IL-6 and increased caveolin facilitates BBB breakdown via an inflammatory-dependent response and downstream activation of TLR4 and p-STAT3 pathway mediated via microglia [29]. Not surprisingly, we found an increase in tight junction protein disruption and luminal irregularity consistent with BBB breakdown on post-SAH day 3. Importantly, we noticed a sharp increase in activated microglia in a perivascular distribution at this same time point, indicating early recruitment as the inflammatory surge rises. This increase was associated with recruitment and infiltration of peripheral myeloid cells. We found that IL-6 blockade prevented BBB disruption and perivascular microglia recruitment on post-SAH day 3 by reducing the pro-inflammatory state. IL-6 blockade also reduced the caveolin increase, thereby stopping the inflammatory recruitment.

Emerging evidence has linked dysregulation of CBF as a contributor to the early BBB breakdown and induction of neuroinflammation [30]. To further test the importance of cerebral autoregulatory perfusion, we measured CBF at 48 h. A significant disruption in right MCA perfusion was seen following SAH. IL-6 KO, and IL-6 blockade prevented this flow disruption through reduced activation of the inflammatory cascade. Maddahi and colleagues noted decreased perfusion 48 h post-SAH in their model and highlighted the correlation with the beginning of the IL-6 surge [31]. Autoregulatory dysfunction may therefore be a key contributor to the early BBB breakdown and inflammatory surge. It likely indicates one of the protective roles we observed with IL-6 blockade and noted benefit of reducing microglia recruitment early. The next logical progression was to look to vasospasm. In our mouse model, we found a vasospasm peak on post-SAH day 5. Both IL-6 blockade and KO prevented the vasospasm development. When IL-6 was reintroduced to the KO mice in the context of SAH, they developed vasospasm in a similar manner to wild-type SAH mice. To tease apart the mechanism, we investigated downstream cascades with specific emphasis on the downstream consequences of the inflammatory cascade. Suzuki and colleagues reported that the TLR4/STAT3 pathway is integral to vasospasm development [32]. The proposed mechanism is a positive feedback loop with IL-6 and may account for the sustained IL-6 levels we found on post-SAH day 5. The graphical abstract shows how IL-6 blockade targets both phases of the IL-6 response.

In our model, we found that IL-6 KO reduced p-STAT3 and TLR4 on post-SAH day 5. This was likely due to a unique microglia phenotype switch, which was prevented by IL-6 blockade. We further selectively targeted p-STAT3 to confirm importance of pathway and found protective benefit against neuroinflammation and oxidative stress. Further projects will help elucidate the role of peripheral macrophages in inducing this switch. We have shown here the early infiltration of peripheral myeloid cells. IL-6 blockade provided similar protective benefit to that seen by IL-6 KO. In addition to reduced vasospasm, the mice with IL-6 blockade had improved modified Garcia scale scores and turn test performance compared to SAH counterparts. Neuroprotection following SAH consists not only of vasospasm treatment, but also prevention of delayed cerebral ischemia (DCI). IL-6 blockade prevented activation of the apoptotic pathway on post-SAH day 7. More selective targeting via the p-STAT3 inhibition confirmed this mechanistic significance. Furthermore, it reduced cell death as measured with Tunel staining on post-SAH day 9. An interesting correlation has been postulated regarding IL-6 surge, vasospasm, and hydrocephalus requiring shunting [33]. We found that IL-6 blockade prevented the increased diameter of the 3^rd^ ventricle after SAH. Again, the inflammatory surge can cause scarring at the arachnoid granulations, which is not present if early inflammatory recruitment is halted. Microglia activation has been associated with both vasospasm and hydrocephalus and is closely regulated by the IL-6 pathway and NLRP3 inflammasome [34].

We had previously reported benefit of NLRP3 inhibition following SAH. In this paper, we found that IL-6 blockade had significant regulation on another arm of the inflammatory spectrum starting on post-SAH day 3 and extending to day 5. Hanafy found that TLR4 knockout in microglia had significant benefit on reducing vasospasm [35]. Likewise reducing IL-6 mitigated the TLR4 response in our model. We have shown that this response is mechanistically linked to the IL-6 surge and that by targeting this surge significant neuroprotective benefit is achieved in a novel mechanistic manner. An area of ongoing investigation is the microglia/peripheral immune crosstalk, which is a topic for future studies. A primary limitation of the study is lack of cerebrospinal fluid collection at extended dates to correlate with timing of delayed cerebral ischemia. Ongoing collaborations are underway to investigate this important human mechanistic time sequence.

## Conclusion

Mechanistic studies regarding the role that IL-6 plays in vasospasm development had up to this point been lacking. Utilizing a host of different assays, inhibition, and knockout animals, we have teased apart the beginning of a mechanistic pathway via the inflammation cascade mediated via STAT3 and peripheral macrophage infiltration. Ongoing work is needed regarding the crosstalk between microglia and peripheral immune cells, which will improve understanding and aid in advancement to first in human trials.

## Data Availability

Not applicable.

## Abbreviations

BBB: Blood brain barrier
CBF: Cerebral blood flow
CV: Cerebral vasospasm
DCI: Delayed cerebral ischemia
IL-6: Interleukin-6
ICA: Internal cerebral artery
KO: Knock out
MCA: Middle cerebral artery
NLRP3: NLR family pyrin domain containing 3
RIPA: Radio-immunoprecipitation assay
STAT3: Signal transducer and activator of transcription 3
SAH: Subarachnoid hemorrhage
TLR4: Toll-like receptor 4
